# First-Principles Investigation of Pressure-Induced Structural Phase Transition and Properties of CsPbF_3_ Polymorphs

**DOI:** 10.1021/acsomega.5c01118

**Published:** 2025-02-26

**Authors:** Paraman Mahalaxmi, Kanimozhi Balakrishnan, Vasu Veerapandy, Nalini Vajeeston, Ponniah Vajeeston

**Affiliations:** † School of Physics, Madurai Kamaraj University, Madurai 625021, India; ‡ Department of Chemistry, Center for Materials Science and Nanotechnology, University of Oslo, Oslo 0371, Norway

## Abstract

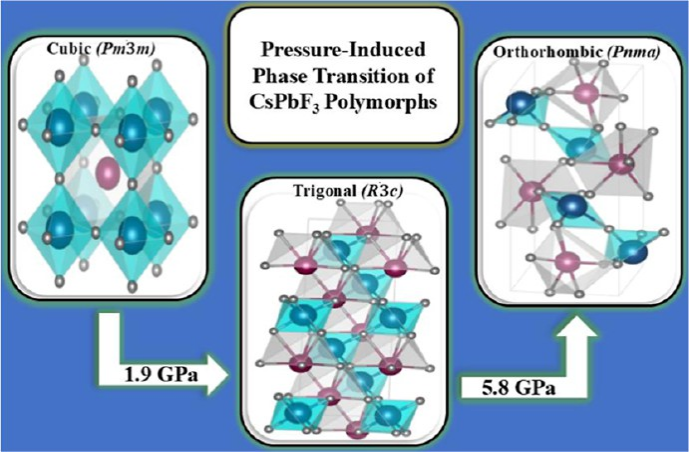

This study presents a first-principles investigation into the high-pressure studies of cesium lead fluoride (CsPbF_3_) polymorph using the Vienna ab initio simulation package (VASP). The CsPbF_3_ with *Pm*3̅*m* symmetry undergoes a pressure-induced structural transition, resulting in two distinct phases: *R*3̅*c* and *Pnma*. The structural stability, electronic structure, and optical properties of the three polymorphs of CsPbF_3_ (*Pm*3̅*m*, *R*3̅*c* and *Pnma*) are investigated using the plane wave pseudopotential method within the framework of density functional theory (DFT). The elastic constants and moduli of these polymorphs were computed and the result confirms that all are mechanically stable. Electronic band structure calculations indicate that all three CsPbF_3_ polymorphs exhibit semiconducting properties with a wide band gap (3–5 eV). The *Pm*3̅*m*, *R*3̅*c* form of CsPbF_3_ has a direct band gap while *Pnma* form has an indirect band gap. The mechanical stability and optical properties of the *R*3̅*c* and the *Pnma* phase of CsPbF_3_ have not been reported in the existing literature. By addressing this gap, this research contributes valuable data and sets the stage for future studies that explore these polymorphs in greater detail and their potential in advanced technological applications.

## 1 Introduction

Perovskite materials are a new class of semiconductor materials that offer a wide range of potential applications, such as sensors, catalytic electrodes in fuel cells, solar cells, lasers, memory devices, optoelectronics, and spintronics.^1−5^ Among perovskite materials inorganic perovskite has been found to exhibit excellent thermal stability compared to hybrid perovskites, which incorporate organic components.^6,7^ The unique properties of inorganic perovskites, such as high bandgap tunability, excellent charge-carrier mobilities, and solution processability, make them attractive for a wide range of applications.^8−10^ Cesium lead fluoride (CsPbF_3_) is a type of halide perovskite that has gained interest in the field of optoelectronics and photovoltaics. It is a wide bandgap (3.68 eV) semiconductor with strong ionic bonding in Cs–F and strong covalent bonding in Pb–F.^11^ The chemical bonding, structural, electronic, and optical properties of cubic CsPbF_3_ (*Pm*3̅*m*) using density functional theory (DFT) were discussed by Murtaza et al.^12^ Their findings concluded that CsPbF_3_ is a direct and wide bandgap semiconductor with a fundamental gap at the R-symmetry point, making it suitable for optoelectronic devices and antireflecting coatings. A recent experimental study by Yan et al.^11^ demonstrated the successful growth of high-quality CsPbF_3_ single crystals using the Bridgman method, investigating their crystal structure, luminescence, and electrical properties. The researchers determined that the crystal structure of CsPbF_3_ remains cubic at room temperature, with a bandgap of 3.68 eV. Another study by Mohammed et al.^13^ examined the structural, electronic, and optical properties of the cubic CsPbF_3_ using DFT. The findings highlight that cubic CsPbF_3_ possesses a wide and direct bandgap, suggesting its potential applications in optical and optoelectronic devices. While prior research has focused solely on the cubic form (*Pm*3̅*m*) of CsPbF_3_, this work aims to introduce and characterize additional polymorphs, providing insights into their structural, mechanical, electronic, and optical properties. Investigating the optical and electrical properties of trigonal (*R*3̅*c*) and orthorhombic (*Pnma*) phases of CsPbF_3_ is crucial for its potential applications in electronic devices such as solar cells and LEDs.^14^ Since perovskite materials have been extensively researched due to their diverse structural phase transitions researchers have been investigating the impact of pressure on metal halide perovskites to enhance their physical and chemical characteristics.^15−17^ Berastegui et al.^18^ conducted an experimental investigation on the structural behavior of cubic CsPbF_3_ (space group *Pm*3̅*m*) as a function of temperature, confirming the presence of a structural phase transition at ∼190 K. This study provided evidence of a transition from the cubic perovskite structure to a rhombohedral distorted perovskite arrangement at low temperatures. The present study demonstrates that cubic CsPbF_3_ undergoes structural phase transitions under pressure, leading to a decrease in symmetry from the *Pm*3̅*m* phase due to octahedral tilting where the cubic structure transitions to a trigonal phase with space group symmetry *R*3̅*c* due to the rotation of PbF_6_ octahedra about the *z*-axis in the cubic Brillouin zone. At higher pressure, a transition occurs from the trigonal phase to an orthorhombic phase with the *Pnma* space group. The pressure-induced structural phase transition from trigonal to orthorhombic symmetry has received significantly less attention than the previous phase. The aim of this study is to provide a comprehensive ab initio investigation of the structural, mechanical, electronic, and optical properties of cubic (*Pm*3̅*m*), trigonal (*R*3̅*c*), and orthorhombic (*Pnma*) polymorphs of CsPbF_3_.

## 2 Results and Discussion

### 2.1 Structural Stability

The stability of CsPbF_3_ perovskite material is influenced by various factors, including the crystal structure transition, octahedral factor, and tolerance factor.^19^ The octahedral factor (μ), a critical requirement is calculated using the following formula
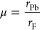
1Here *r*_Pb_ and *r*_F_ are the ionic radii of lead (1.19 Å) and fluoride (1.33 Å) respectively.^20^ The perovskite structures typically remain stable when the octahedral factor ranges from 0.4420 to 0.8950.^21^ CsPbF_3_ perovskite has an octahedral factor of 0.8947, placing it near the upper limit of the stable range.^11,22^ An extensively used and effective geometric ratio is the Goldschmidt tolerance factor (*t*_G_), which can be defined as follows
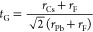
2Here *r*_Cs_ is the ionic radii of cesium (1.88 Å). The Goldschmidt tolerance factor is commonly employed to study distorted perovskites.^19,23^ The tolerance factor has been accepted widely as a key criterion for forming the perovskite structure. This factor evaluates whether the Cs-site cation can adeptly fit within the cavities of the PbF_3_ framework. A stable perovskite structure with high absorption capabilities is established within the tolerance factor range of 0.8–1.0.^24^ CsPbF_3_ perovskite possesses a tolerance factor of 0.841, residing near the threshold of the stable perovskite structure.^11,22^ This suggests that CsPbF_3_ perovskite might exhibit distinctive characteristics due to its nearly stable nature.^22^

First-principles calculations were performed using structural data from the Materials Project to predict the equilibrium crystal structures of halide perovskites. The results largely agree with experimental structures, demonstrating that the Materials Project is effective in forecasting the structural properties of these materials. The accuracy of predictions improves significantly when a broader range of existing structural information for similar chemical formulas, such as those in the ABX_3_ (A is a monovalent cation, B is any one of the divalent metal and X is a halide anion) category is considered. The reliability of these predictions is influenced by the number of input structures used in the calculations. Selecting appropriate input structures for the ABX_3_ composition from the Materials Project database is a tedious process that requires substantial computational resources. Many phases display similar structural types, and in some cases, the positional parameters of certain atoms differ only slightly.^25^ As a result, these structures often converge to similar configurations during full geometry optimization, leading to the disregard of these subtle variations. In total, nearly 40 distinct structural types with unique arrangements have been identified in this composition, as detailed in Table S1 of the Supporting Information.

**Figure 1:**
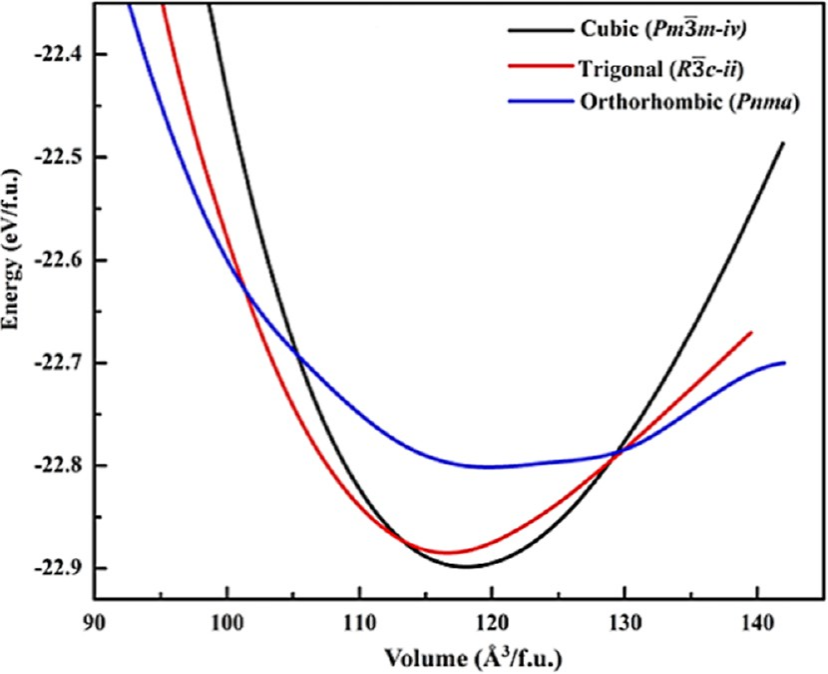
Total energy vs volume curve of CsPbF_3_ polymorphs.

By fitting energy–volume curves, 13 low-energy polymorphs of CsPbF_3_ were identified shown in Figure S1 of the Supporting Information. Polymorphs with higher energy levels were excluded from further consideration due to their reduced stability. This highlights the importance of energy considerations in assessing the stability of the material. Figure 1 shows the analysis of the relationship between energy and volume for CsPbF_3_ polymorphs. The energy–volume curve provides valuable insights into stability differences among these crystal structures. The configuration with the lowest energy represents the most stable state. The cubic CsPbF_3_ polymorph (*Pm*3̅*m-iv*, #221) showed the lowest energy (−22.89 eV/f.u.) at its equilibrium volume of 118.05 Å^3^/f.u., while the orthorhombic CsPbF_3_ polymorph (*Pnma*, #62) exhibited the highest energy (−22.80 eV/f.u.) among the considered phases. This indicates that the cubic crystal system with the space group *Pm*3̅*m* is the most stable configuration of all. Further support for this observation comes from the work of Yan et al.,^11^ who identified the CsPbF_3_ crystal structure as cubic at room temperature. Figure 1 shows that at a lower volume, the cubic CsPbF_3_ (*Pm*3̅*m-iv*) curve intersects with the trigonal CsPbF_3_ (*R*3̅*c-ii*, #167) curve, indicating the structural transition from cubic to trigonal. With increased volume compression, the trigonal CsPbF_3_ (*R*3̅*c-ii*) curve intersects with the orthorhombic CsPbF_3_ (*Pnma*) curve, signifying a transformation from the trigonal form to the least stable orthorhombic state. This signifies the intricate structural changes experienced by CsPbF_3_ under varying pressure conditions.^26^ Thus, out of 13 polymorphs of CsPbF_3_, only three polymorphs [i.e., cubic (*Pm*3̅*m-iv*) trigonal (*R*3̅*c-ii*), and orthorhombic (*Pnma*)] were considered for further investigation.

**Figure 2:**
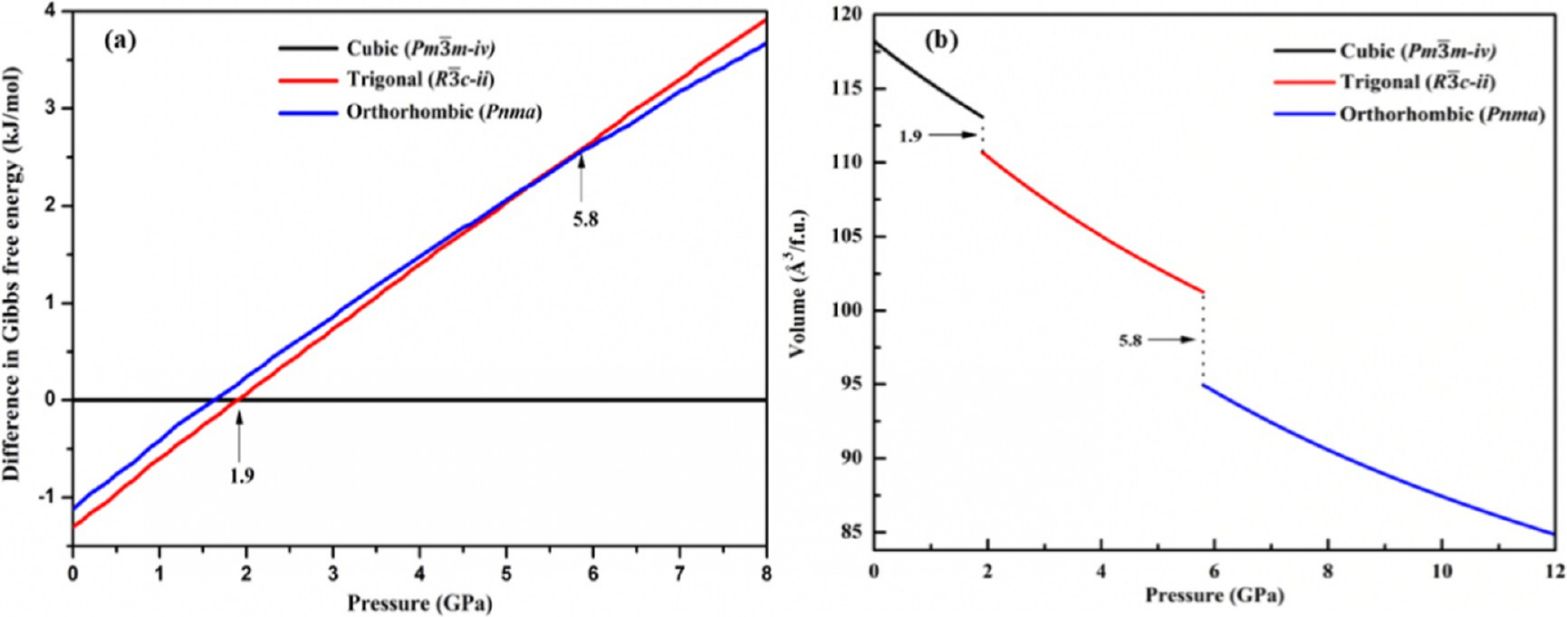
(a) Stabilities of phases involved in the structural transition relative to cubic (*Pm*3̅*m*) phase as a function of pressure. (b) Pressure versus volume relation for CsPbF_3_ polymorphs.

To provide a clear picture of structural transition, Figure 2a shows the Gibbs energy difference between the polymorphs of CsPbF_3_ relative to cubic (*Pm*3̅*m*) as a function of pressure. The difference in Gibbs free energy (*G* = *H* – *TS*) between phases as a function of pressure, includes contributions from both enthalpy (*H*) and entropy (*S*). These values are obtained from first-principles simulations that account for temperature and pressure dependence. At constant temperature (0 K), phase transitions occur at pressures where the Gibbs free energies of two phases are equal (Δ*G* = 0). These points indicate thermodynamic equilibrium between the phases, marking the transition pressure.^26,27^ The transition pressure is marked by arrows at the transition points. Figure 2b presents the volume as a function of pressure for each phase. The transition from *Pm*3̅*m-iv* to *R*3̅*c-ii* occurs at 1.9 GPa resulting in a small volume shrinkage of 2.37 Å^3^/f.u. Subsequently, the transition from *R*3̅*c-ii* to *Pnma* takes place at 5.8 GPa and is accompanied by a volume shrinkage of 6.3 Å^3^/f.u. These calculations are specifically applicable to stoichiometric compounds at low temperatures. The pressure-induced structural transition of CsPbF_3_ has not been previously addressed in the literature, underscoring the significance of this research in providing new insights to the field.

**Figure 3:**
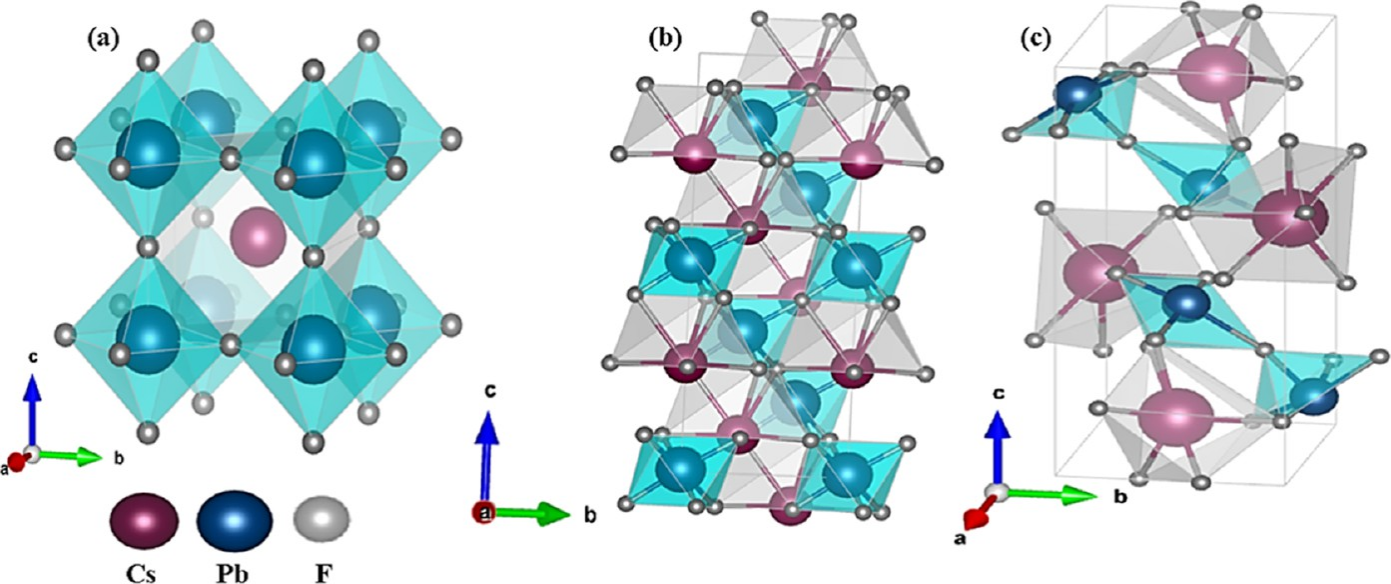
Optimized crystal structure of (a) cubic (*Pm*3̅*m*), (b) trigonal (*R*3̅*c*) and (c) orthorhombic (*Pnma*) CsPbF_3_ polymorphs.

In Figure 3, the optimized crystal structures of the three CsPbF_3_ polymorphs are shown. The cubic CsPbF_3_ system consists of PbF_6_ octahedra surrounding a central Pb atom with Cs atoms positioned at the corners of the cubic structure (Figure 3a). CsPbF_3_ initially exists in a cubic phase, transitioning to a trigonal phase as shown in Figure 3b, and then to an orthorhombic phase at high pressure. This results in a structural distortion evident in the octahedra, as depicted in Figure 3c. The transition from the cubic (*Pm*3̅*m*) phase to the trigonal (*R*3̅*c*) phase occurs due to the rotation of PbF_6_ octahedra around specific crystallographic axes. This rotation disrupts the high symmetry of the cubic structure, leading to a reorganization of the octahedral framework. As a result, the material reduces its total energy and effectively manages the strain caused by external pressure. Octahedral tilting is a well-recognized mechanism in perovskite materials that aids in stabilizing the crystal structure under various conditions.^28^ To investigate this structural transition, the angular displacements of Pb–F–Pb bond angles and related changes in bond lengths are essential. These geometric modifications are vital for understanding how the material adapts structurally to minimize energy and accommodate any lattice strain that arises from external factors such as pressure or temperature changes. The transition from the trigonal phase (*R*3̅*c*) phase to the orthorhombic (*Pnma*) phase introduce complex crystalline changes. This transition involves cooperative octahedral tilting along multiple axes, resulting in significant lattice strain and a reduction in symmetry. These distortions manifest as rotations and displacements that alter the effective bond angles and distances within the PbF_6_ octahedra. Distinct patterns, including in-phase and out-of-phase tilts of the octahedra, are linked to pressure-induced changes in lattice parameters.^28^

Table 1 outlines the positional and lattice parameters obtained from the DFT calculations at both the equilibrium and phase transition points, along with the values determined experimentally and theoretically. The calculated lattice constants for the cubic polymorph is (*a* = 4.908 Å) align closely with the experimentally confirmed cubic phase (*a* = 4.800 Å).^18^ The trigonal polymorph with space group *R*3̅*c* features a rhombohedral lattice structure within the trigonal crystal system. The calculated lattice parameters for this phase are *a* = 6.966 Å and *c* = 16.599 Å, showing good agreement with the theoretically calculated values for trigonal (*R*3̅*c*) CsPbF_3_ reported by Smith et al.^31^ which are (*a* = 6.809 Å, *c* = 16.306 Å). The lattice parameters for the orthorhombic polymorph of CsPbF_3_ and the remaining structural parameters, including Wyckoff positions and their corresponding sites, are detailed in Table 1.

**Table 1:** Lattice and Positional Parameters of CsPbF_3_ Polymorphs at Equilibrium and Phase Transition Point

S. no.	polymorphs of CsPbF_3_	lattice constant (Å) (at equilibrium)	lattice constant (Å) (at phase transition point)	atom	site	*x*	*y*	*z*	atom	site	*x*	*y*	*z*
				(at equilibrium)					(at phase transition point)				
1.	cubic (*Pm*3̅*m-iv*)	*a* = 4.908	*a* = 4.825	Cs(1)	1*b*	0.5	0.5	0.5	Cs(1)	1*b*	0.5	0.5	0.5
		*a* = 4.800^18^		Pb(1)	1*a*	0.0	0.0	0.0	Pb(1)	1*a*	0.0	0.0	0.0
		*a* = 4.973^29^		F(1)	3*d*	0.5	0.0	0.0	F(1)	3*d*	0.5	0.0	0.0
		*a* = 4.806^30^											
2.	trigonal (*R*3̅*c-ii*)	*a* = 6.966	*a* = 6.863 *c* = 16.247	Cs(1)	2*a*	0.25	0.25	0.25	Cs(1)	2*a*	0.25	0.25	0.25
		*c* = 16.599		Pb(1)	2*b*	0.50	0.50	0.50	Pb(1)	2*b*	0.50	0.50	0.50
		*a* = 6.809^31^		F(1)	6*e*	0.69	0.80	0.25	F(1)	6*e*	0.69	0.80	0.25
		*c* = 16.306^31^											
3.	orthorhombic (*Pnma*)	*a* = 6.819	*a* = 6.331	Cs(1)	4*c*	0.49	0.25	0.13	Cs(1)	4*c*	0.48	0.25	0.14
		*b* = 4.851	*b* = 4.724	Pb(1)	4*c*	0.48	0.25	0.62	Pb(1)	4*c*	0.52	0.25	0.61
		*c* = 14.312	*c* = 13.451	F(1)	4*c*	0.29	0.25	0.75	F(1)	4*c*	0.31	0.25	0.75
				F(2)	4*c*	0.45	0.75	0.63	F(2)	4*c*	0.48	0.75	0.63
				F(3)	4*c*	0.20	0.25	0.54	F(3)	4*c*	0.21	0.25	0.54

### 2.2 Mechanical Properties

The mechanical stability of CsPbF_3_ polymorphs was thoroughly analyzed to gain insights into their behavior under applied forces. In particular, the elastic constants, which describe how materials respond to deformations caused by external forces, were studied in detail. To assess the mechanical stability of CsPbF_3_ polymorphs, these elastic constants were determined, which helps in predicting deformations under applied forces. The linear elastic constants are represented by a symmetric matrix, comprising multiple components. The number of independent elastic constants depends on the symmetry of the space group of the material being studied.^32^ For the cubic CsPbF_3_ polymorph, the elastic properties were characterized using three independent elastic components (*C*_11_, *C*_12_, and *C*_44_), which reflect responses to specific deformations along different crystallographic directions.^33^ The trigonal CsPbF_3_ polymorph has seven independent elastic components (*C*_11_, *C*_12_, *C*_13_, *C*_14_, *C*_33_, *C*_44_, and *C*_66_) and orthorhombic CsPbF_3_ polymorph has nine independent elastic components (*C*_11_, *C*_12_, *C*_13_, *C*_22_, *C*_23_, *C*_33_, *C*_44_, *C*_55_ and *C*_66_).^25,34^ These have been calculated and summarized in Table 2. For the structures to be stable, the elastic constants must satisfy the Born stability criteria which are a set of conditions on the elastic constants (*C*_ij_) related to the second-order changes in the internal energy of a crystal under deformation.^33^ The Born stability criteria of elastic constants for cubic, trigonal, and orthorhombic systems are given as follows^25,33,34^
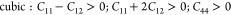

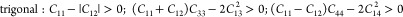

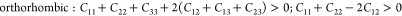

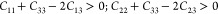


**Table 2:** Calculated Elastic Constants (*C*_*ij*_), of CsPbF_3_ Polymorphs in GPa

S.no.	CsPbF_3_ polymorphs	*C*_11_	*C*_12_	*C*_13_	*C*_14_	*C*_22_	*C*_23_	*C*_33_	*C*_44_	*C*_55_	*C*_66_
1	cubic (*Pm*3̅*m-iv*)	79.25 75.12^29^ 97.91^35^	18.57 16.73^29^ 20.36^35^						10.97 9.69^29^ 6.33^35^		
2	trigonal (*R*3̅*c-ii*)	53.74	33.80	26.43	0.63			40.61	11.93		9.96
3	orthorhombic (*Pnma*)	49.71	6.67	11.39		17.91	7.49	19.03	11.93	8.28	9.35

The results revealed that all three polymorphs of CsPbF_3_ exhibited stable phases, satisfying the Born stability criteria necessary for mechanical stability.

The dominance of the *C*_11_ elastic constant indicates greater resistance to deformation when stress is applied along the *x*-axis. In trigonal configuration, the larger value of *C*_12_ suggests that the trigonal phase is more responsive to changes in shape when subjected to shear stress. The comparatively smaller *C*_33_ value in the orthorhombic polymorph indicates lower stiffness when stress is applied along the *z*-direction, indicating stronger bonding within the *xy*-plane relative to the out-of-plane directions. The independent elastic constants obtained from each structure were utilized in the Voigt–Reuss–Hill method to calculate elastic parameters such as the bulk modulus (*B*), Young’s modulus (*E*), and shear modulus (*G*).^36−38^

**Figure 4:**
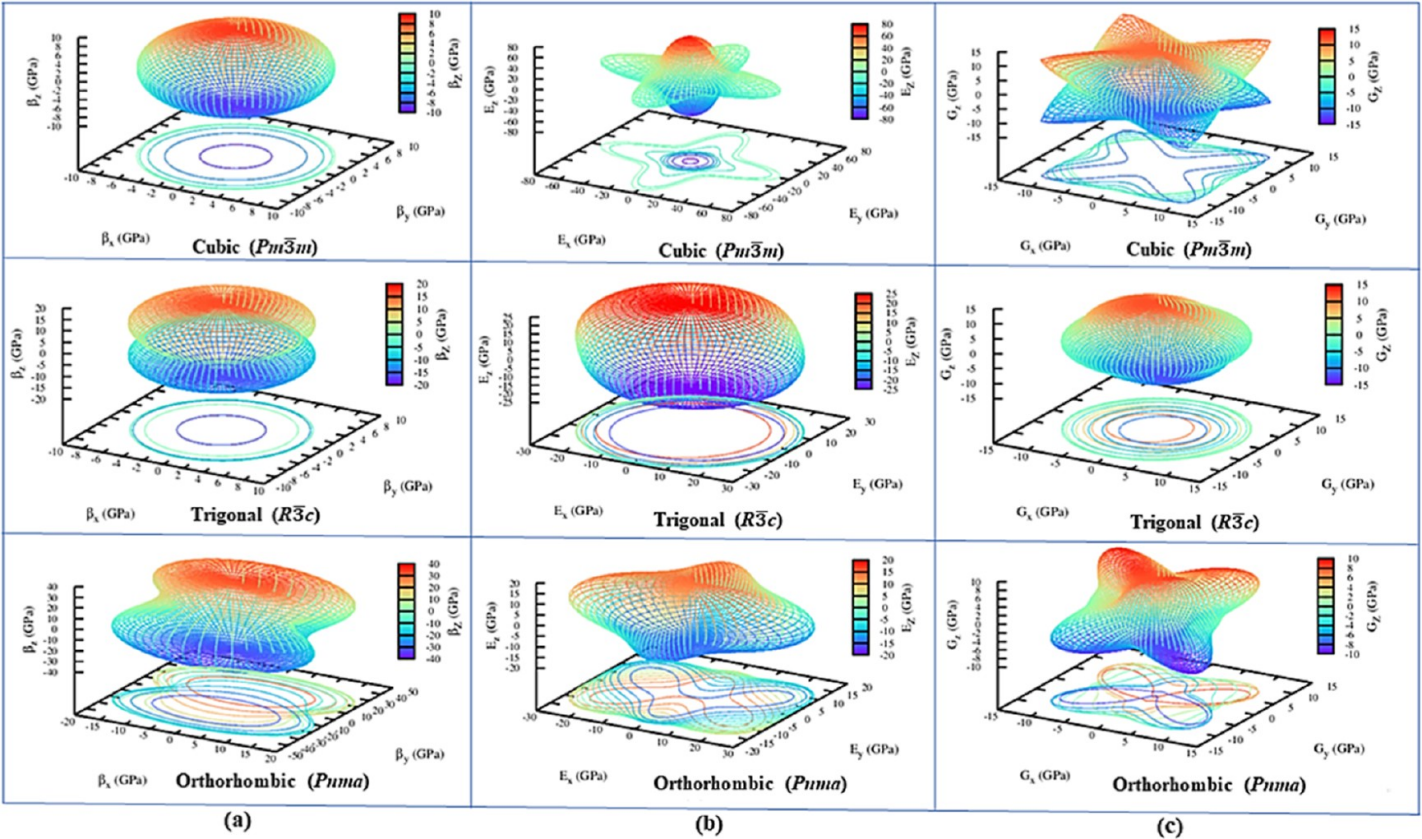
Spatial dependence of the (a) bulk modulus (*B*) (b) Young’s modulus (*E*) and (c) shear modulus (*G*) of cubic (*Pm*3̅*m*), trigonal (*R*3̅*c*), and orthorhombic (*Pnma*).

Table 3 represents the calculated bulk modulus (*B*), Young’s modulus (*E*), shear modulus (*G*), Poisson ratio (ν) and Pugh’s ratio (*B*/*G*) of CsPbF_3_ polymorphs by applying Hill’s approximation which is averaging Voigt’s and Reuss’s results. The bulk modulus relates to how incompressible a material is. Higher bulk modulus signifies that the material can withstand changes in volume under pressure without deforming significantly. The cubic phase exhibits a higher bulk modulus due to its symmetrical and dense atomic arrangement, making it more resistant to compression. The orthorhombic phase, with its lower bulk modulus, demonstrates a greater tendency to compress under applied pressure. The cubic form of CsPbF_3_ exhibits the highest Young’s modulus among the polymorphs, indicating that it is relatively stiff and resistant to deformation under tensile stress. The trigonal phase has a lower Young’s modulus than the cubic phase, indicating it is more flexible and can deform more easily under stress. This flexibility could be beneficial in certain applications, such as in flexible electronics or optical elements that need to undergo structural changes without breaking. The orthorhombic phase displays the lowest Young’s modulus, suggesting it is the least stiff and most prone to deformation under applied stress. Shear modulus measures the response of a material to shear stress and is crucial for understanding how a material will perform under torsional forces. The cubic structure has a higher shear modulus indicating that it can effectively resist shape deformations when subjected to shear forces, making it reliable in structural applications where such forces are present. The low shear modulus in the orthorhombic phase suggests that it is more susceptible to deformation under shear stress. The elastic anisotropy describes how material properties, such as stress and strain, depend on the direction of applied forces. The cubic phase has relatively high symmetry, resulting in isotropic elastic properties. While it may still exhibit some anisotropy depending on the specific material characteristics, its elastic moduli are generally uniform in all directions due to its face-centered cubic lattice structure. This makes the cubic polymorph reliable for applications where uniform mechanical properties are essential, such as in structural components and electronic devices. The trigonal polymorph exhibits very low elastic anisotropy. This means that its mechanical response to applied stress is less directionally dependent, resulting in more uniform behavior across different loading directions. This characteristic can be advantageous in applications needing flexibility or resistance to multidirectional loading, like flexible electronics. The uniform elasticity can simplify modeling and fabrication processes, making it easier to predict performance. The orthorhombic phase displays higher elastic anisotropy compared to both cubic and trigonal forms. This is due to the lower symmetry in its crystal structure, which leads to significant directional dependence on its mechanical properties. Such variations mean that the orthorhombic phase may exhibit different strengths and rigidity when forces are applied along different crystallographic axes. The investigation into the anisotropic effect on shear and Young’s moduli emphasizes their crucial role in reflecting the directional variations in the mechanical properties of anisotropic materials. A material is considered isotropic if it displays a symmetrical circular shape, indicating uniform mechanical response regardless of orientation within the 2*D* plane, while deviations from this circular symmetry reveal anisotropic characteristics. The noncircular contours observed in cubic and orthorhombic polymorphs of CsPbF_3_ as seen in Figure 4b,c indicate anisotropy, depicting the directional dependency of CsPbF_3_ in mechanical responses. The calculated elastic anisotropy for the cubic and orthorhombic polymorphs is 1.35 and 1.66, respectively, indicating their directional dependence. The elastic anisotropy for the trigonal polymorph is 0.14, suggesting isotropic behavior, as supported by the circular contours of *G* and *E* in Figure 4b,c. The analysis indicates that both *G* and *Y* moduli are highly anisotropic in all directions for the orthorhombic polymorph.

**Table 3:** Calculated Bulk Modulus (*B*), Young’s Modulus (*E*), Shear Modulus (*G*), Poisson Ratio (ν), Pugh’s Ratio (*B*/*G*), Elastic Anisotropy (*A*), and Cauchy’s Pressure (*P*_c_) of CsPbF_3_ Polymorphs

S.no.	CsPbF_3_ polymorphs	*B* (GPa)	*E* (GPa)	*G* (GPa)	ν	*B*/*G*	*A*	*P*_c_ (GPa)
1	cubic (*Pm*3̅*m-iv*)	38.80 36.19^29^ 26.50^35^	43.87 40.36^29^ 34.83^35^	16.73 15.35^29^ 13.53^35^	0.31	2.32 2.35^29^ 2.00^35^	1.35	7.6
2	trigonal (*R*3̅*c-ii*)	34.97	29.28	10.76	0.36	3.25	0.14	21.9
3	orthorhombic (*Pnma*)	13.84	20.50	8.18	0.25	1.69	1.66	–1.3

The nature of atomic bonding and ductility is elucidated using Cauchy’s pressure (*P*_c_). The calculated *P*_c_ values for the cubic and trigonal structures are 7.6 and 21.9 GPa, respectively, suggesting the presence of covalent bonds. In contrast, the orthorhombic structure exhibits a *P*_c_ value of −1.3 GPa, signifying metallic bonds due to its negative value. Pugh’s ratio is a key parameter that aids in the assessment of material toughness, brittleness and mechanical behavior, offering valuable guidance in material selection, and predicting the response of the material to external loads. The characterization of the materials’ brittle or ductile attributes involves the utilization of Pugh’s ratio (*B*/*G*). According to Pugh’s empirical relationship, when the ratio of the bulk modulus (*B*) to the shear modulus (*G*) exceeds 1.75, the material is considered ductile, and if the ratio is less than 1.75, the material is categorized as brittle.^39^ In the current analysis, the cubic and trigonal polymorphs of CsPbF_3_ exhibit Pugh’s ratios greater than 1.75, indicating their ductile nature. The orthorhombic polymorph of CsPbF_3_ does not meet the condition of Pugh’s ratio, hence it is considered brittle. The evaluation of Poisson’s ratio (ν) is imperative in assessing the intrinsic toughness of the material. If ν exceeds 0.26, the material displays relative toughness, whereas ν value below 0.26 suggests brittleness.^40^ The cubic and trigonal polymorphs manifest Poisson’s ratios greater than 0.26, signifying relative toughness, while the orthorhombic polymorph does not fulfill the condition of Poisson’s ratio, thereby indicating brittleness. These characteristics suggest that the cubic and trigonal polymorphs of CsPbF_3_ are highly suitable for applications in flexible printed electronic devices. The optimized mechanical parameters, including elastic constants and moduli of elasticity, for the cubic polymorph align well with previous theoretical literature, as detailed in Tables 2 and 3. This comprehensive analysis has significant implications for understanding the mechanical behavior and stability of CsPbF_3_ polymorphs, offering valuable contributions to the field of materials science and engineering.

### 2.3 Electronic Structure

**Figure 5:**
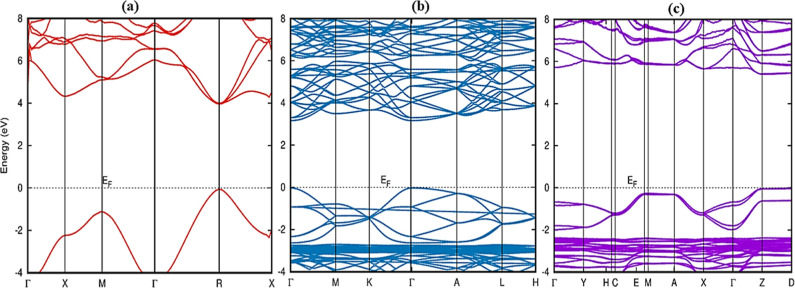
Band structure of (a) cubic (*Pm*3̅*m-iv*), (b) trigonal (*R*3̅*c-ii*) and (c) orthorhombic (*Pnma*) CsPbF_3_ polymorphs.

To obtain comprehensive insights into the electronic structures of cubic, trigonal, and orthorhombic polymorphs of CsPbF_3_, the band structures were estimated using HSE06 functionals. The band structures for cubic (*Pm*3̅*m*), trigonal (*R*3̅*c*), and orthorhombic (*Pnma*) CsPbF_3_ were analyzed along the paths Γ, X, M, Γ, R, X, Γ, M, K, Γ, A, L, and Γ, Y, H, C, EM, A, X, Γ, Z respectively, illustrated in Figure 5 with the top of the valence band (VB) set to 0 eV for all calculations. For the cubic phase of CsPbF_3_, band structure calculations revealed that the valence band maximum (VBM) and conduction band minimum (CBM) were both situated at point R, yielding a direct bandgap. Being a direct band gap material means that electrons can transition between the valence band and conduction band directly without requiring a change in momentum. This characteristic is particularly favorable for light-emitting applications (such as LEDs and laser diodes) because it promotes efficient photon emission. The calculated bandgap value (*E*_g_) value obtained using the HSE06 functional is 3.96 eV, which is comparable with the experimentally determined value of 3.68 eV.^11^ Mohammed et al.,^13^ calculated *E*_g_ for cubic CsPbF_3_ using the GW method and found it to be approximately 4.05 eV. Khan et al.^29^ used generalized gradient approximation with the PBE approach and calculated *E*_g_ around 2.98 eV for cubic CsPbF_3_.

In the trigonal phase of CsPbF_3_, the VBM, and CBM are found at point Γ, indicating a direct energy gap of 3.24 eV. This suggests that it can efficiently emit light, although it would emit at a different wavelength than the cubic phase due to the lower band gap. As depicted in Figure 5c, the VBM for the orthorhombic CsPbF_3_ phase is positioned at point Z, while the CBM is located at a different point, resulting in an indirect *E*_g_ of 5.83 eV. This is due to the structural distortion in the octahedral structures. Being an indirect band makes it less efficient for optical applications compared to the cubic and trigonal phases. However, the wider band gap orthorhombic phase can find applications in UV-responsive devices. No experimental bandgap value has been identified in the literature for the CsPbF_3_ trigonal and orthorhombic phases. The calculated and previously reported bandgap values for CsPbF_3_ polymorphs are displayed in Table 4.

**Table 4:** Calculated Band Gap Values (HSE-06) of CsPbF_3_ Polymorphs and Their Corresponding Band Gap Types Are Listed

CsPbF_3_ polymorphs	cubic (*Pm*3̅*m-iv*)	trigonal (*R*3̅*c-ii*)	orthorhombic (*Pnma*)
band gap (eV)	3.96	3.24	5.41
	3.68^11^		
	4.05^13^		
	2.98^29^		
band gap type	direct	direct	indirect

**Figure 6:**
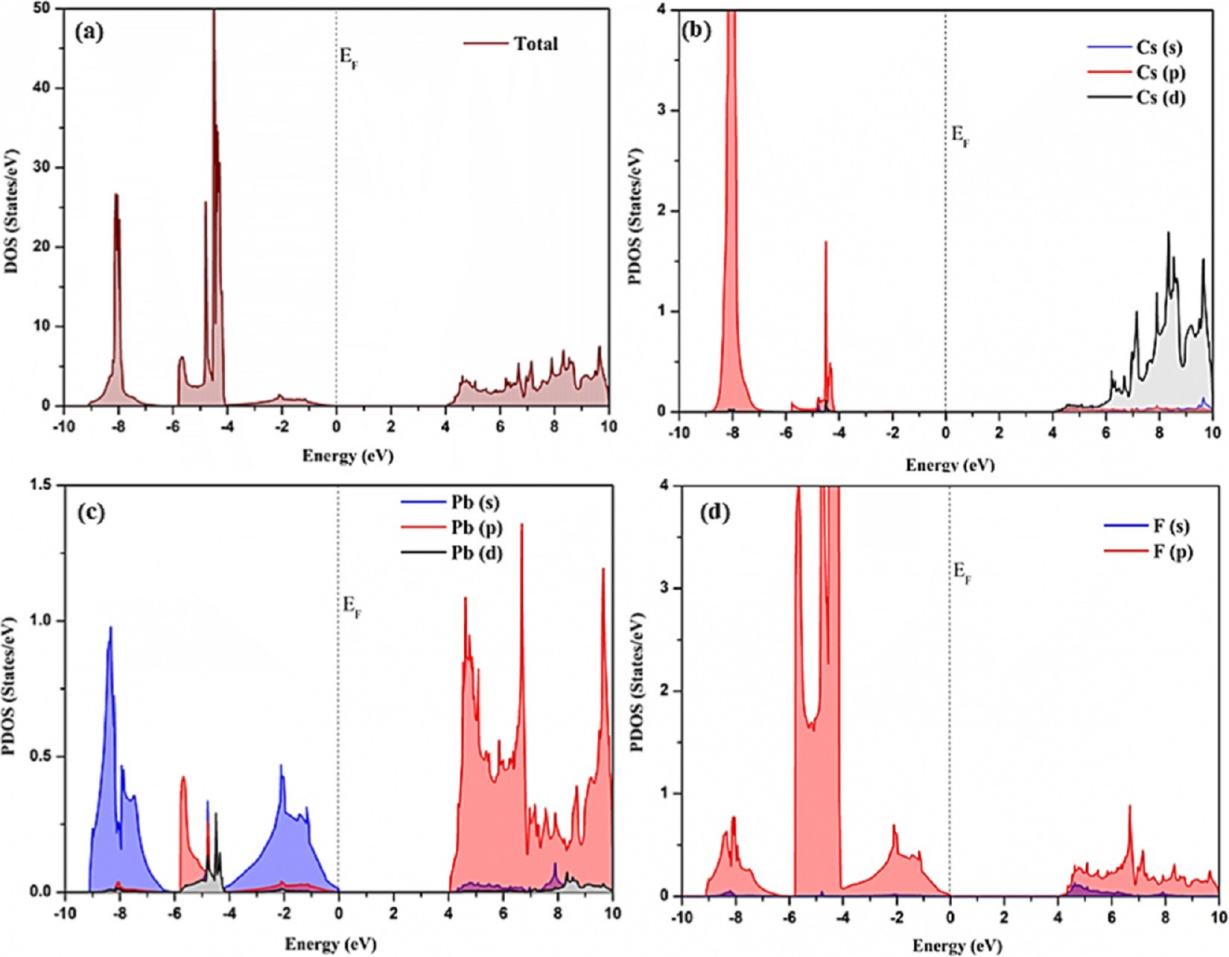
Calculated (a) total density of states of cubic CsPbF_3_ (b) projected density of states of Cs orbitals (c) projected density of states of Pb orbitals (d) projected density of states of F orbitals.

**Figure 7:**
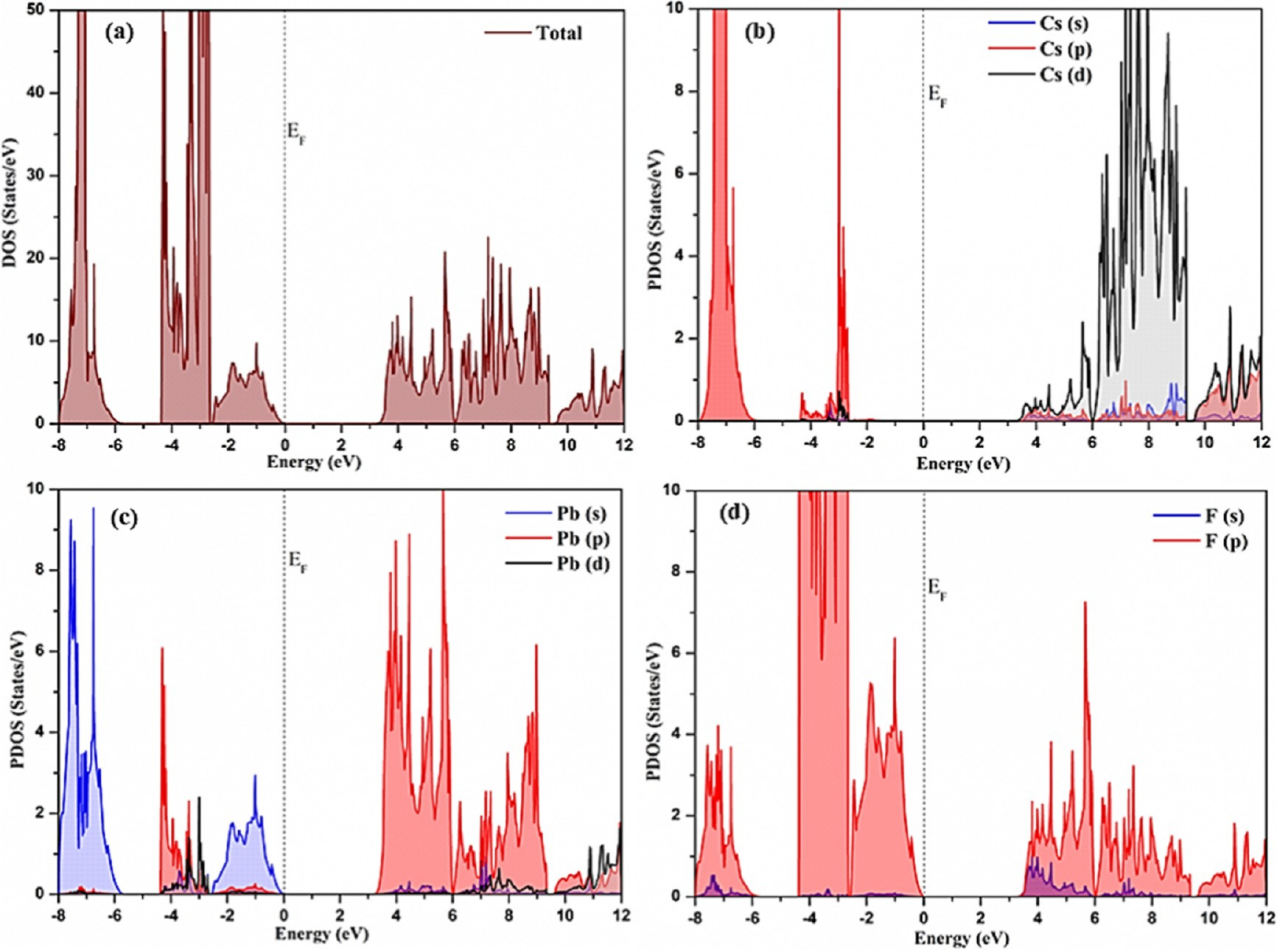
Calculated (a) total density of states of trigonal CsPbF_3_, (b) projected density of states of Cs orbitals,, (c) projected density of states of Pb orbitals, (d) projected density of states of F orbitals.

**Figure 8:**
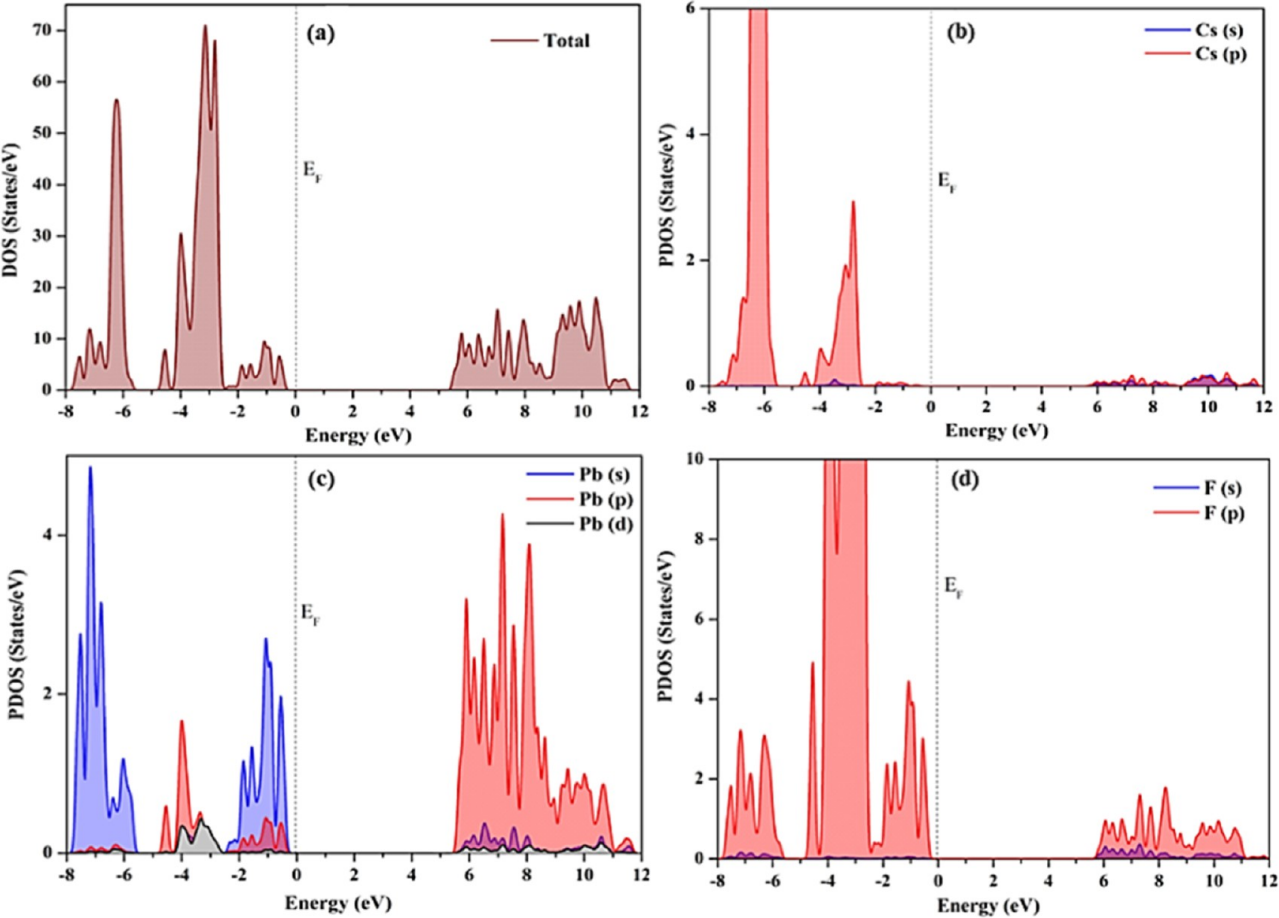
Calculated (a) total density of states of orthorhombic CsPbF_3_, (b) projected density of states of Cs orbitals, (c) projected density of states of Pb orbitals, (d) projected density of states of F orbitals.

To gain a comprehensive understanding of the band structure of CsPbF_3_ polymorphs, the total density of states (TDOS) and the partial density of states (PDOS) are plotted. The TDOS and PDOS for cubic, trigonal, and orthorhombic CsPbF_3_ are illustrated in Figures 6–8 respectively. Within these plots, the black vertical dashed line positioned at 0 eV signifies the Fermi energy (*E*_F_) level. The PDOS of cubic CsPbF_3_ is illustrated in Figure 6 indicates that the valence band is primarily influenced by the Cs 5p, Pb 6s, and F 2p electronic states, with minor involvement from Pb 6p and 5d orbitals near the *E*_F_. The conduction band is predominantly formed by Cs 4d and Pb 6p orbitals with minor involvement of F 2p orbitals. The Pb 6s state and the Pb 6p demonstrate a significant role in forming the VBM and CBM respectively. In the PDOS analysis, the Pb 6s electrons play a pivotal role in absorbing photon energy, while the Pb 6p electrons are crucial for generating photocurrent. This result is in good agreement with the previous ab initio calculations.^12,41^

The PDOS shown in Figure 7 corresponding trigonal phase reveals that the VBM is predominantly composed of the 5p orbitals of Cs along the 6s orbitals of Pb, and the 2p orbitals of F with notable hybridization observed between them indicating strong interactions between these elements. The CBM is primarily contributed by the 4d orbitals of Cs, and the 6p orbitals of Pb along with the 2p orbitals of F. A minor contribution from the 5d orbitals of Pb and the 2s orbitals of F are also seen. The PDOS illustrated in Figure 8 for the orthorhombic phase of CsPbF_3_ suggests the prominent contribution of the Cs 5p, Pb 6s, and F 2p states to the valence band, with minor involvement from Pb 6p and 5d orbitals near the *E*_F_. The conduction band predominantly arises from Pb 6p and F 2p orbitals with minor involvement of Pb 5d and F 2s orbitals.

### 2.4 Optical Properties

**Figure 9:**
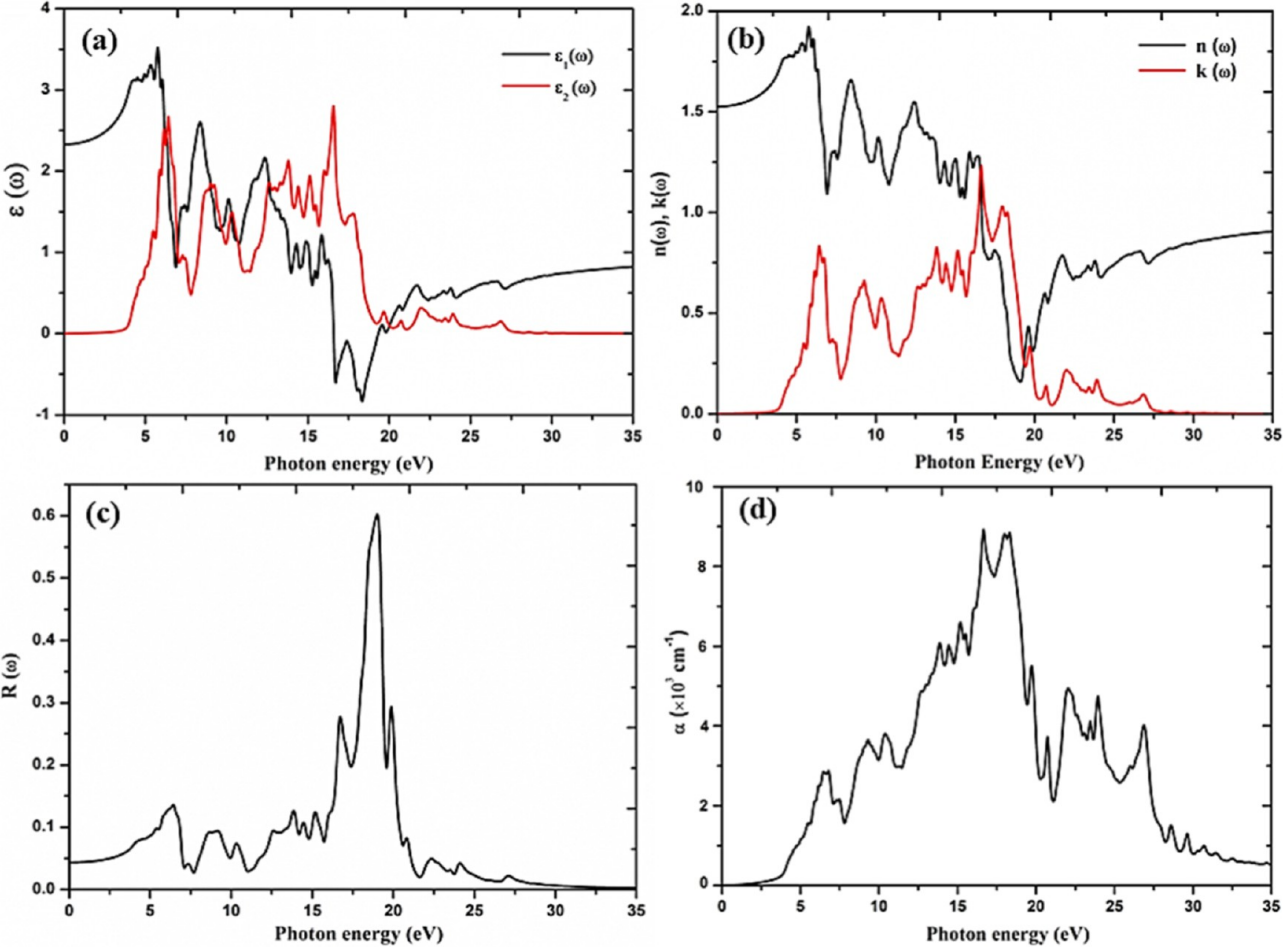
(a) Calculated real ε_1_(ω) and imaginary ε_2_(ω) parts of the dielectric functions, (b) refractive index *n*(ω) and extinction coefficient *k*(ω) spectra, (c) reflectivity spectra and (d) optical absorption coefficient as a function of the photon energy of cubic CsPbF_3_.

The optical properties of perovskites are crucial for investigating their potential use in optoelectronic devices. Cubic CsPbF_3_ is highly valued in optics due to its exceptional characteristics, which include a wide optical band gap, minimal optical loss, and excellent transparency within the visible and near-infrared regions.^13,29^ The optical properties are computed using the hybrid functional HSE-06, providing a detailed description of optical constants such as the dielectric function ε(ω), including both real and imaginary parts, refractive index *n*(ω), extinction coefficient *k*(ω), reflectivity *R*(ω), and absorption coefficient α(ω). The complex dielectric (ε) = ε_1_(ω) + *i*ε_2_(ω) is an important optical parameter that describes the complete response of any material to the applied electromagnetic photons.^12^ The dielectric function of a material has two components: the real component ε_1_(ω) and the imaginary component ε_2_(ω). In the context of solar cells, ε_1_(ω) indicates the ability of the cell to store energy, while ε_2_(ω) determines its absorption capabilities.^13^ The imaginary component of a material is linked to its electronic band structure and its ability to absorb light. The real component is derived from the imaginary part, reflecting the optical and electronic response of the material. Knowing these components makes it possible to calculate the optical properties of the material. Using specific computational methods, the real and imaginary components of the dielectric function were computed for a range of incident photon energies. The resulting frequency-dependent optical properties of cubic CsPbF_3_ were then calculated, and the values are depicted in Figure 9. The dielectric function is calculated based on the incident photon energy in the range 0–35 eV. From Figure 9a, the threshold point (onset) in the spectra of ε_2_(ω) is found at 3.78 eV, which is due to the transition of electrons from mixed Pb 6s and F 2p states in the valence band (VB) to the Pb 6p states in the conduction band (CB) near the Fermi level, as seen in Figure 6.^41^

The threshold energy corresponds to the fundamental absorption edge, which signifies the optical transition between the highest valence and lowest conduction bands. This point is closely related to the electronic bandgap of the compound, which is 3.96 eV. Following the threshold energy, the curve of the imaginary part increases rapidly, and the presence of multiple peaks refers to band-to-band transitions. The peaks around 5.88 and 8.37 eV originate from the electronic transitions from F 2p and Pb 6s in the valence band to the Pb 6p state in the conduction band.^12^ In Figure 9a, the zero-frequency limit ε_1_(0), which is the electronic part of the static dielectric constant is depicted. Our calculated ε_1_(0) for cubic CsPbF_3_ is 2.326. From its zero-frequency limit, it starts increasing and reaches the maximum value of 3.535 at 5.88 eV, and then it decreases with noticeable variations. Also, it goes below zero in the range of 16.66–19.38 eV. In this range, the incident photon beam is completely attenuated. In this negative energy region, the real part of the dielectric function exhibits metallic behavior, while the non-negative region represents dielectric behavior. This detailed understanding of the dielectric function and its components provides valuable insights into the optical properties of the CsPbF_3_ perovskite, which is essential for evaluating its potential applications in photovoltaic devices.

Figure 9b shows the relationship between the refractive index, denoted as *n*(ω), and its extinction coefficient, represented as *k*(ω) of cubic CsPbF_3_. It is interesting to note that *k*(ω) follows a similar trend to ε_2_(ω). Looking at the spectrum of *k*(ω), we see that the medium’s maximum absorption occurs at 15.95 eV. The calculated refractive index for the compound is 1.53. It is evident from Figure 9b that the refractive index of the material increases from its zero-frequency limit and reaches a maximum value of 1.912 at 5.8 eV. After the maximum value, it starts decreasing and goes below unity after 16.72 eV. When the refractive index is less than unity, it indicates that the group velocity (*V*_g_ = *c*/*n*) of the incident radiation is greater than the speed of light *c*.^42^ It indicates that the group velocity shifts to a negative domain, and the nature of the medium changes from linear to nonlinear. In other words, the material becomes superluminal for high-energy photons.^43^ The refractive index is used to calculate the optical reflectivity of the material.^44^ The calculated reflectivity of cubic CsPbF_3_ perovskites has been presented in Figure 9c. The zero-frequency reflectivity *R*(0) value is 0.02589 for CsPbF_3_. The zero-frequency reflectivity is 2.6%, and it remains almost the same up to 3 eV. The low reflectivity in the near-infrared and visible energy range indicates that the material is transparent in this range. Therefore, it can be used as an antireflective coating in this part of the energy spectrum. Beyond 4 eV, the reflectivity increases sharply and reaches a maximum of 60% at 19.04 eV, with noticeably smaller peaks at other energies. The maximum reflectivity occurs where the value of ε_1_ goes below zero, as seen in Figure 9a,c. The computed absorption coefficient (α) for cubic CsPbF_3_ is presented in Figure 9d. The absorption spectrum predicts how many photons of various energies are absorbed and is directly connected to the fundamental energy gap. Photons with energies lower than the fundamental energy gap are transmitted, while photons with equal and higher energies are absorbed. The cubic CsPbF_3_ is highly effective in the UV region, with low optical absorption observed in the visible range, allowing efficient transmission of photon energy in this range. Figure 9d shows that absorption starts around 3 eV and then increases to its maximum value of 9 × 10^3^ cm^–1^ at 16.6 eV. Figure 9a,c reveal that absorption is higher for energies at which ε_1_(ω) is negative, and maximum absorption occurs where ε_1_(ω) has a maximum negative value. The significant values of α(ω), cover a wide energy range, approximately (3–30 eV), making the cubic CsPbF_3_ perovskite a promising material for various optical and optoelectronic devices in the high-energy visible light and low ultraviolet ranges. These calculated optical properties of cubic CsPbF_3_ are in good agreement with the existing literature values.^12^

**Figure 10:**
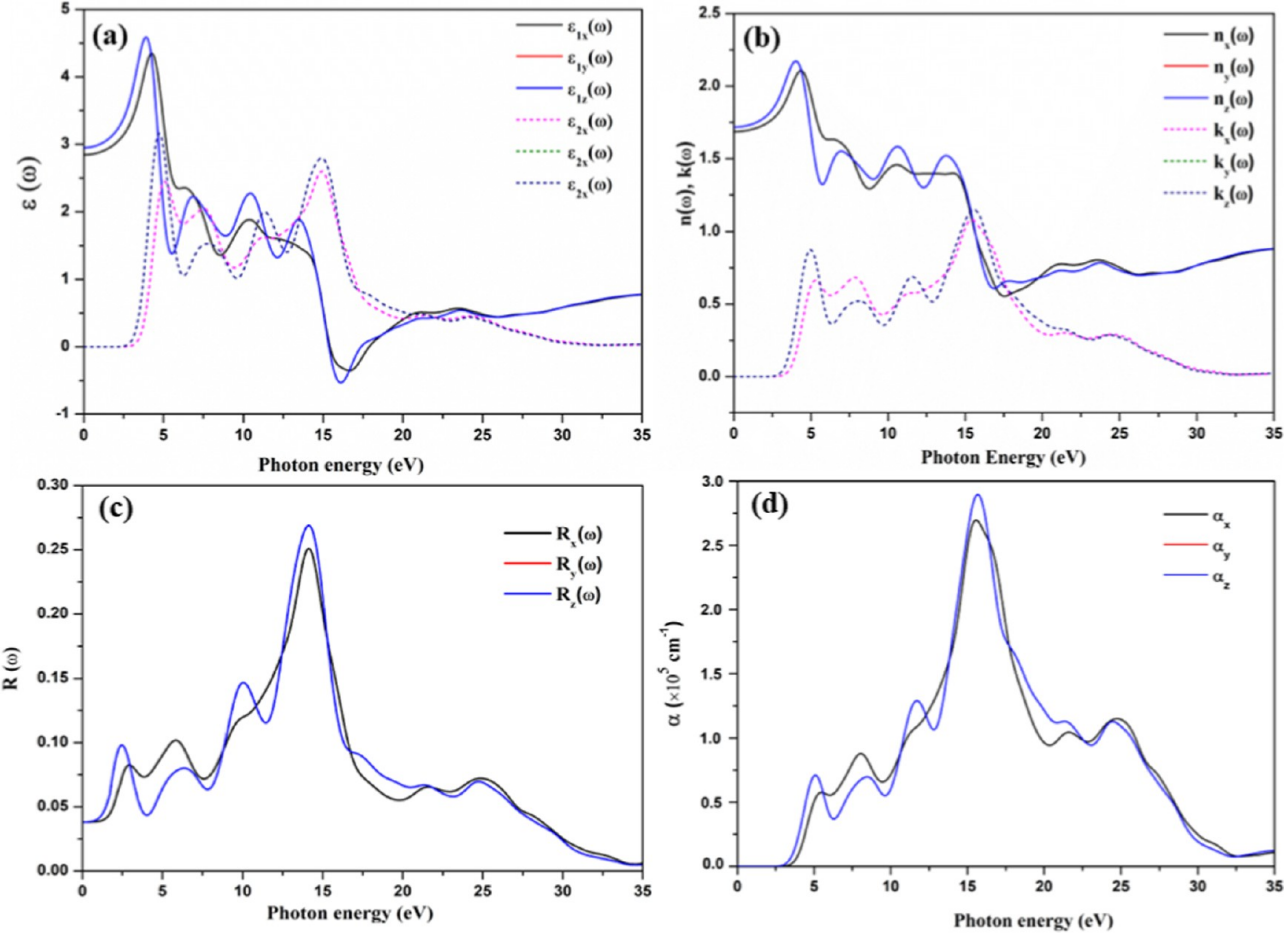
(a) Calculated real ε_1_(ω) and imaginary ε_2_(ω) parts of the dielectric functions, (b) refractive index *n*(ω) and extinction coefficient *k*(ω) spectra, (c) reflectivity spectra and (d) optical absorption coefficient as a function of the photon energy for trigonal CsPbF_3_.

**Figure 11:**
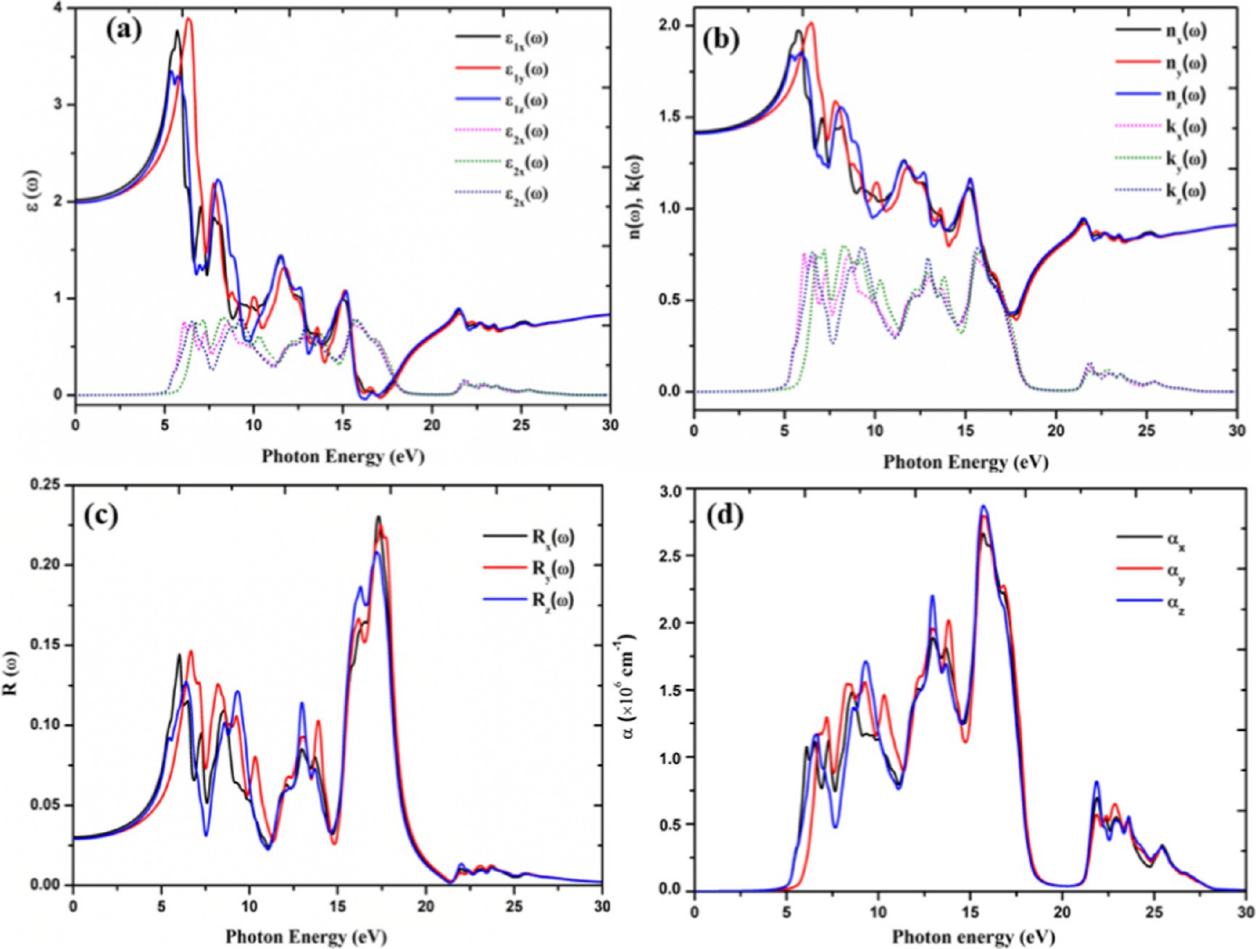
(a) Calculated real ε_1_(ω) and imaginary ε_2_(ω) parts of the dielectric functions, (b) refractive index *n*(ω) and extinction coefficient *k*(ω) spectra, (c) reflectivity spectra and (d) optical absorption coefficient as a function of the photon energy for orthorhombic CsPbF_3_.

The optical properties of a trigonal phase of CsPbF_3_, as shown in Figure 10, exhibit anisotropic behavior due to its symmetry characteristics. This anisotropy determines how the material interects with light and varies depending on crystallographic direction. Figure 10 shows that the optical responses along *y* and *z* axes are identical, indicating that interactions with light in both the *y* and *z* directions yield the same values. In Figure 10a, the electronic component of the static dielectric constant, denoted as ε_1_(0), is illustrated, showing an averaged value of 2.9 at zero frequency. As the frequency increases, ε_1_(ω) along *x*, *y* and *z* axes begins to rise, reaching a maximum value of 4.6 at approximately 4.5 eV. Beyond this point, the dielectric constant declines and exhibits significant fluctuations. The dielectric constant drops below zero in the energy range of 15.22–16.13 eV, indicating that the incident photon beam is completely absorbed in this interval. The spectra of the imaginary part of the dielectric function, ε_2*x*_(ω), indicates a threshold point at an energy of 3.32 eV. This threshold energy is closely linked to the fundamental absorption edge of the material, which corresponds to a bandgap of 3.24 eV. Beyond this threshold, the curve for the imaginary part of the dielectric function rises sharply, revealing multiple peaks associated with transitions between different energy bands within the material. Figure 10b illustrates the relationship between the refractive index *n*(ω), and the extinction coefficient *k*(ω), of trigonal CsPbF_3_. The extinction coefficient shows a trend that closely resembles that of the imaginary part of the dielectric function, ε_2_(ω). Analyzing the *k*(ω), we observe that the material reaches its peak absorption at an energy of 15.64 eV. The calculated refractive index for the trigonal CsPbF_3_ is approximately 1.7. This higher refractive index leads to stronger light–matter interactions, which enhances its applicability in optical devices. Figure 10c presents data on the reflectivity of trigonal CsPbF_3_ perovskites. The zero-frequency reflectivity, *R*(0), is recorded at 0.045, which corresponds to a reflectivity of 4.5%. This low reflectivity remains relatively stable up to about 3 eV. Such low values in the near-infrared and visible regions indicates significant transparency in these energy ranges, suggesting potential applications in optoelectronic devices. Above 10 eV, the reflectivity increases sharply, reaching a maximum of 28% at 14.7 eV, with noticeably smaller peaks at other energies. These anisotropic optical properties is essential for potential applications in optoelectronic devices. The computed absorption coefficient (α) for trigonal CsPbF_3_ is displayed in Figure 10d, which provides insight into the absorption characteristics across various energy levels. From Figure 10d, absorption begins at approximately 3 eV and gradually increases, peaking at a value of 2.8 × 10^5^ cm^–1^ at 16.6 eV. The optical properties of orthorhombic CsPbF_3_ were calculated and depicted in Figure 11. The optical properties of the orthorhombic phase display significant anisotropy due to its lower symmetry and distinct optical response along different crystallographic directions. The dielectric function is calculated using the incident photon energy from 0 to 30 eV. From Figure 11a, the threshold energy corresponds ε_1*x*_(ω) and ε_1*z*_ (ω) is 4.77 eV. The threshold point in the spectra of ε_1*y*_ (ω) is found at 5.54 eV. This point is closely related to the fundamental bandgap of the compound, which is 5.41 eV. Following the threshold energy, the curve of the imaginary part increases rapidly, and the presence of multiple peaks refers to band-to-band transitions. In the Figure 11a, the average calculated zero-frequency limit ε_1_(0) is 2. From the zero-frequency limit, ε_1*y*_ (ω) has the maximum value of 3.835 at 6 eV, and then it decreases with noticeable variations. Figure 11b shows the relationship between *n*(ω), and *k*(ω) of orthorhombic CsPbF_3_. The calculated refractive index for the compound is 1.41. It is evident from Figure 11b that the refractive index *n*_*y*_ of the material increases from its zero-frequency limit and reaches a maximum value of 2.01 at 5.3 eV.

The calculated reflectivity of orthorhombic CsPbF_3_ perovskite has been presented in Figure 11c. The zero-frequency reflectivity *R*(0) value is 0.03. The maximum reflectivity is observed in the UV (16–19 eV) region. The computed absorption coefficient (α) for orthorhombic CsPbF_3_ is presented in Figure 11d. The orthorhombic CsPbF_3_ is also highly effective in the UV region allowing efficient transmission of photon energy in this range. The absorption coefficients transmit within the visible range. Figure 11d shows that absorption starts around 5.44 eV and then increases to its maximum value of 2.8 × 10^6^ cm^–1^ at 17 eV. The optical anisotropy in the orthorhombic phase can be leveraged for applications such as optical waveguides, polarizers, and devices requiring tailored light manipulation based on polarization. The CsPbF_3_ polymorphs exhibit high absorption coefficients in the UV range, making it highly efficient for UV-responsive devices. This effective UV absorption is critical for developing technologies that require sensitivity to UV light. When compared with other perovskite materials like CsPbBr_3_ which exhibits strong absorption coefficients in the visible range but is limited in the UV domain.^45^ This limitation restricts its applications in technologies that require effective UV light absorption, reducing its versatility in the broader spectrum of optical devices. Similarly, MAPbI_3_ shows strong absorption in the visible region, making it suitable for applications in solar cells.^46^ However, like CsPbBr_3_, it is less effective in UV absorption, which limits its utility in UV-dependent applications. The inorganic composition of CsPbF_3_ gives it excellent thermal stability, allowing it to withstand high temperatures without significant degradation. This property is essential for devices exposed to varying thermal conditions, ensuring long-term reliability, whereas MAPbI_3_, being a hybrid organic–inorganic perovskite, is less stable under thermal and environmental stress. CsPbF_3_ is highly effective at absorbing UV light, making it suitable for UV-sensitive detection technologies and more advanced optoelectronic devices that require interaction with UV light.

## 3 Conclusion

This study presents a comprehensive analysis of the structural, mechanical, electronic, and optical properties of CsPbF_3_ polymorphs with the space group *Pm*3̅*m*, *R*3̅*c*, and *Pnma* through first-principles calculations. Under ambient conditions, CsPbF_3_ crystallizes in a cubic phase (*Pm*3̅*m*) and at a pressure of 1.9 GPa, it transitions to a trigonal structure with space group *R*3̅*c*. At a higher pressure of 5.8 GPa, the *R*3̅*c* phase further transforms into an orthorhombic structure with space group *Pnma*. The lattice constant values for cubic (*Pm*3̅*m*) CsPbF_3_ polymorph aligns well with the experimental results. The cubic structure exhibits a wide direct bandgap of 3.96 eV. Mechanical analysis reveals that the cubic (*Pm*3̅*m*) CsPbF_3_ polymorph has the highest *C*_11_ value among the three phases, indicating its strong mechanical stability and resistance to deformation. The lattice parameters for trigonal (*R*3̅*c*) polymorph align well with the theoretical results. The trigonal (*R*3̅*c*) phase shows a moderate band gap of 3.24 eV (direct), which allows it to absorb incident light and emit in the visible range. Its higher refractive index (1.8) enhances light–matter interactions, making it suitable for optical devices. The moderate stiffness of this phase provides a balance between flexibility and rigidity, making it effective for applications where some deformation is acceptable, such as flexible electronics. The orthorhombic (*Pnma*) phase of CsPbF_3_ has the lowest *C*_11_ value among the three phases, signifying that it is the least stiff and most susceptible to deformation under applied stress. This orthorhombic structure displays a wide indirect band gap of 5.41 eV with a high absorption coefficient of 3 × 10^6^ cm^–1^, which allows it to capture UV light to effectively. The ability to induce structural transitions under pressure facilitates tunable electronic and optical properties, potentially beneficial for applications such as tunable lasers, sensors, and photovoltaics. Future work should focus on experimental validation of the theoretical predictions and exploration of the lesser-studied trigonal and orthorhombic forms of CsPbF_3_ to fully harness the potential of this versatile material.

## 4 Computational Detail

First-principles calculations based on density functional theory (DFT) were conducted using the Vienna ab initio simulation package (VASP).^47^ The interactions between ions and electrons in all elemental components were modeled using the projector augmented wave (PAW) approach within VASP.^48^ Pseudopotentials were applied with the following valence states: Cs (5s^2^5p^6^6s^1^), Pb (6s^2^5d^10^6p^2^), and F (2s^2^2p^5^). The Perdew, Burke, and Ernzerhof (PBE) parametrized generalized gradient approximation (GGA) incorporating a self-interaction correction method was chosen for the exchange–correlation functional.^49^ The conjugate-gradient algorithm, with a force convergence criterion of less than 10^–3^ eV Å^–1^ was utilized to determine ground-state geometries by minimizing stresses and Hellman–Feynman forces. Throughout all relaxation processes, Brillouin zone integration was performed using a Gaussian broadening of 0.1 eV. In order to span a wide range of energetically accessible crystal structures, unit-cell volume and shape as well as atomic positions were relaxed simultaneously in a series of calculations made with progressively increasing precision. After completing the relaxations, a final high accuracy calculation of the total energy was performed, ensuring convergence of *k*-point and plane-wave cutoff energy. The crystal structure optimization, including lattice parameters, site coordinates, and Wyckoff positions, was visualized using VESTA, a tool specializing in three-dimensional visualization.^50^ A Monkhorst–pack grid scheme was utilized for *k*-point sampling in the Brillouin zone for the various polymorphs.^51^ The optimization process achieved convergence with an energy cutoff set at 500 eV. The *k*-point grid densities used for each polymorph in our calculations and presented in Table S2 of Supporting Information. Elastic constants were determined using the finite strain method with the VASPKIT postprocessing tool.^52^ The method employed to calculate the mechanical moduli (Young’s modulus E and shear modulus *G*) are given in Supporting Information. Band structures and optical spectra were obtained through the application of the Heyd–Scuseria–Ernzerhof (HSE-06) screened hybrid functional.^53^

## Supporting Information Available

List of structure types involved in the selection of CsPbF_3_ polymorphs, energy volume curve of selected CsPbF_3_ polymorphs, *k*-point grids of CsPbF_3_ polymorphs and mechanical moduli calculations (PDF)
