# Supplementary material for: First-Principles Investigation of Pressure-Induced Structural Phase Transition and Properties of CsPbF3 Polymorphs
Source: ACS Omega. 2025 Feb 26;10(9):9793–807. doi: 10.1021/acsomega.5c01118 (PMC11904671; doi:10.1021/acsomega.5c01118)
Supplement: Supplementary file 1 — ao5c01118_si_001.pdf [file ao5c01118_si_001.pdf]

Supporting information of "First-principles investigation of Pressure-induced Structural Phase Transition and Properties of $CsPbF_3$ Polymorphs"

Paraman Mahalaxmi [1], Kanimozhi Balakrishnan[1], Vasu Veerapandy[1], Nalini Vajeeston[2] and Ponniah Vajeeston[2*]

[1]*School of Physics, Madurai Kamaraj University, Madurai 625021, India.*

[2]*Department of Chemistry, Center for Materials Science and Nanotechnology, University of Oslo, Oslo 0371, Norway.*

1. The selection of $CsPbF_3$ polymorphs involved input structure types in the selection of ABX_3 polymorphs are listed in Table S1.

Table S1: List of structure types involved in the selection of $CsPbF_3$ polymorphs

mp-20282_$CsPbF_3$	mp-675524_$CsPbCl_3$	mp-998298_$CsSnF_3$	mp-616378_$CsSnI_3$	mp-998193_$RbSnF_3$
mp-5811_$CsPbF_3$	mp-23037_$CsPbCl_3$	mp-1247749_$CsSnBr_3$	mp-21043_$RbPbF_3$	mp-998620_$RbSnF_3$
mp-567681_$CsPbBr_3$	mp-675022_$CsPbCl_3$	mp-27214_$CsSnBr_3$	mp-2767545_$RbPbF_3$	mp-1198796_$RbSnF_3$
mp-567629_$CsPbBr_3$	mp-540839_$CsPbI_3$	mp-27349_$CsSnCl_3$	mp-1540546_$RbPbF_3$	mp-998604_$RbPbCl_3$
mp-1014168_$CsPbBr_3$	mp-1120768_$CsPbI_3$	mp-1070375_$CsSnCl_3$	mp-674508_$RbPbF_3$	mp-998605_$RbPbCl_3$
mp-1120725_$CsPbBr_3$	mp-1069538_$CsPbI_3$	mp-614013_$CsSnI_3$	mp-1179712_$RbPbF_3$	mp-28774_$KSnF_3$
mp-600089_$CsPbBr_3$	mp-1105915_$CsSnF_3$	mp-27381_$CsSnI_3$	mp-605350_$RbPbF_3$	mp-1097014_$KSnBr_3$
mp-1539667_$CsPbCl_3$	mp-998457_$CsSnF_3$	mp-568570_$CsSnI_3$	mp-1173075_$RbPbF_3$	mp-1097015_$KSnI_3$

2. Total energy vs volume curve of selected $CsPbF_3$ polymorphs

* Corresponding author

E-mail address: vajeeston.ponniah@kjemi.uio.no; https://folk.universitetetioslo.no/ponniahv/

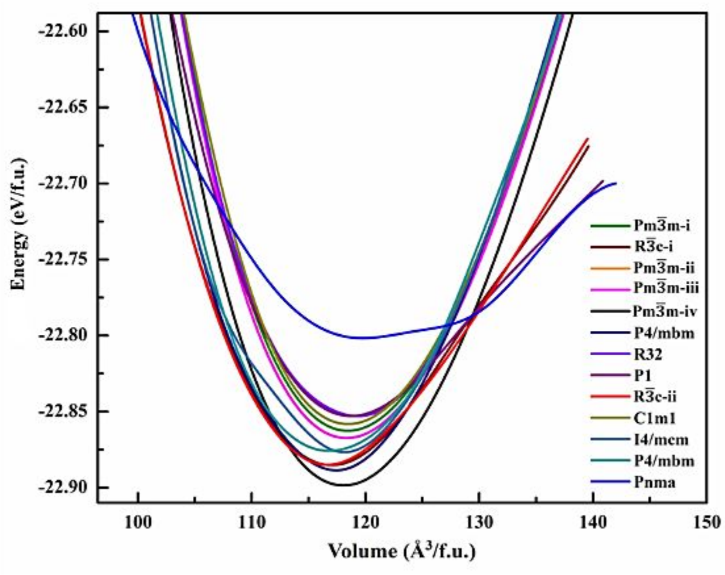

Figure S1. Total energy vs volume curve of selected 13 $CsPbF_3$ polymorphs

The selected polymorphs shown in Figure S1 crystallize into various crystal structures, with four exhibiting a cubic configuration, three trigonal systems, three tetragonal systems, and each in an orthorhombic, triclinic, and monoclinic arrangement. Among these polymorphs of $CsPbF_3$, only three polymorphs (i.e. cubic (*Pm*$\overline{3}$*m-iv*) trigonal (*R*$\overline{3}$*c-ii*), and orthorhombic (*Pnma*)) were considered for further investigations in this work.

3. k-point Grids: We have specified the k-point grid densities used for each polymorph in our calculations in Table S2.

Table S2. Used K points for the HSE, DOS and Optical calculations

S.No	Polymorphs	HSE	DOS	Optics
1.	Cubic (*Pm*$\overline{3}$*m-iv*)	3×3×3	9×9×9	12×12×12
2.	Trigonal (*R*$\overline{3}$*c-ii*)	2×2×1	6×6×3	8×8×4
3.	Orthorhombic (*Pnma*)	5×3×1	10×6×3	12×8×4

4. Mechanical Moduli Calculations: We have used the stress-strain approach, performed finite deformations, and computed the respective moduli based on variations in energy due to applied strains. The method employed to calculate the mechanical moduli (Young's modulus E and shear modulus G) are as follows

Young's Modulus (E): First, optimize the structure fully (cell parameters and atomic positions) and apply small strains to the optimized structure. Then calculate stress-strain relationships.

For a cubic system, E can be derived from the three independent elastic constants using:

$$E = [C_{11} - C_{12} + 3C_{44}] [C_{11} + 2C_{12}] / [2C_{11} + 3C_{12} + C_{44}]$$

Similarly, the Shear Modulus (G) is calculated from elastic constants:

$$G = (C_{11} - C_{12} + 3C_{44}) / 5$$

Similarly, there are six independent elastic constants for trigonal crystals exhibiting specific symmetry properties; for orthorhombic material, there are nine independent elastic constants. The appropriate formulas have been applied to compute Young's modulus and Shear modulus.
